# Interstrain Cooperation in Meningococcal Biofilms: Role of Autotransporters NalP and AutA

**DOI:** 10.3389/fmicb.2017.00434

**Published:** 2017-03-22

**Authors:** Jesús Pérez-Ortega, Antonio Rodríguez, Eduardo Ribes, Jan Tommassen, Jesús Arenas

**Affiliations:** Section Molecular Microbiology, Department of Biology, Utrecht University Utrecht, Netherlands

**Keywords:** *Neisseria*, biofilms, bacterial interactions, AutA, NalP, NHBA, IgA protease

## Abstract

*Neisseria meningitidis* (*Nm*) and *Neisseria lactamica* (*Nl*) are commensal bacteria that live in the human nasopharynx, where they form microcolonies. In contrast to *Nl, Nm* occasionally causes blood and/or meningitis infection with often fatal consequences. Here, we studied interactions between neisserial strains during biofilm formation. Fluorescent strains were engineered and analyzed for growth in single- and dual-strain biofilms with confocal laser-scanning microscopy. Different strains of diverse *Neisseria* species formed microcolonies of different sizes and morphologies. Pair-wise combinations of two invasive *Nm* strains and one *Nm* carrier isolate showed that these strains can coexist in spite of the fact that they produce toxins to combat congeners. This lack of competition was even observed when the biofilms were formed under nutrient limitation and can be explained by the observation that the separate microcolonies within mixed biofilms are mostly lineage specific. However, these microcolonies showed different levels of interaction. The coexistence of two strains was also observed in mixed biofilms of *Nm* and *Nl* strains. Inactivation of the autotransporter NalP, which prevents the release of the heparin-binding antigen NHBA and the α-peptide of IgA protease from the cell surface, and/or the production of autotransporter AutA increased interactions between microcolonies, as evidenced by close contacts between microcolonies on the substratum. Qualitative and quantitative analysis revealed an altered spatial distribution of each strain in mixed biofilms with consequences for the biomass, biofilm architecture and bacterial viability depending on the synthesis of NalP and AutA, the expression of which is prone to phase variation. Being in a consortium resulted in some cases in commensalism and cooperative behavior, which promoted attachment to the substratum or increased survival, possibly as result of the shared use of the biofilm matrix. We hypothesize that *Nm* strains can cooperate during host colonization, but, possibly, the different capacities of the microcolonies of each strain to resist the host's defenses limits the long-term coexistence of strains in the host.

## Introduction

The genus *Neisseria* includes bacterial species that colonize mucosal surfaces in humans, e.g., *N. lactamica* (*Nl*), and the pathogenic *N. meningitidis* (*Nm*), which, like *Nl*, inhabits the upper respiratory tract, and *N. gonorrhoeae* (*Ng*), which infects the genitourinary tract. *Nm* forms microcolonies in the nasopharynx (Sim et al., 2000). Such microbial communities are similar to biofilms and offer protection against the host's immune response and other adverse conditions (Costerton et al., 1995). Biofilms are defined as communities of microorganisms attached to a surface and embedded in a self-produced extracellular matrix (Costerton et al., 1995). Multiple factors influence biofilm formation in *Nm*, such as the capsule, type IV pili, surface-exposed proteins, and extracellular polymeric substances of which extracellular DNA (eDNA) can be an important component (Arenas and Tommassen, 2017). Depending on the clonal complex (cc), the bacteria use two different strategies to initiate biofilm formation, i.e., either dependent or independent of eDNA (Lappann et al., 2010). In the former strategy, eDNA is key to initial attachment and structure stabilization. These processes are facilitated by cell-surface-exposed proteins that attach the cells to eDNA via electrostatic interactions, a process that may also occur in other bacterial species (Arenas et al., 2013a; Arenas and Tommassen, 2017).

NalP, IgA protease, AutA, and AutB are autotransporters (AT) involved in the initiation of biofilm formation (Arenas et al., 2013a, 2015a, 2016). ATs are modular proteins constituted of a translocator domain that is inserted into the outer membrane, thereby allowing the transport of the fused passenger domain to the cell surface. After translocation, the passenger may remain attached to the cell or be released into the milieu after proteolytic processing (Grijpstra et al., 2013). NalP (a.k.a. AspA) is a serine protease whose autoproteolytic processing results in its release from the cell surface (Turner et al., 2002). However, temporarily, NalP remains attached at the cell surface by an N-terminal lipid anchor, and in this position, it cleaves several ATs, including IgA protease, and surface-exposed lipoproteins, including the heparin-binding antigen NHBA (van Ulsen et al., 2003; Serruto et al., 2010; Roussel-Jazédé et al., 2013). In addition, NalP cleaves the complement factor C3, thus protecting *Nm* from complement activation (Del Tordello et al., 2014). The passenger of IgA protease consists of two separate domains, the protease domain and the α-peptide, which are connected via a small γ-peptide (Pohlner et al., 1987). The protease domain is released into the extracellular milieu via autocatalytic processing (Pohlner et al., 1987), but it may also be released connected to the α-peptide after cleavage by NalP (van Ulsen et al., 2003; Roussel-Jazédé et al., 2014).

The synthesis of NalP is prone to phase variation, which is a stochastic turning on and off of gene expression (Saunders et al., 2000). In the absence of NalP, the α-peptide of IgA protease (αP) usually remains attached at the cell-surface (Roussel-Jazédé et al., 2014). This αP contains nuclear localization signals, which are positively charged, arginine-rich peptide segments (Pohlner et al., 1995) that bind eDNA and therefore, when present at the cell surface, they increase initiation of biofilm formation. NHBA also contains an arginine-rich region, which is responsible for binding heparin (Serruto et al., 2010) and presumably also for the demonstrated DNA-binding capacity of the protein (Arenas et al., 2013a). The binding of NHBA to eDNA is relevant for the initiation of biofilm formation, at least in strains following an eDNA-dependent strategy (Arenas et al., 2013a). NalP cleavage releases a fragment of NHBA including the arginine-rich region, which has been shown to increase endothelial permeability by inducing the internalization of adherens junction proteins (Casellato et al., 2014). However, this cleavage is not completely effective since full-length NHBA molecules remain detectable at the cell surface in the presence of NalP (Serruto et al., 2010). This uncleaved form plays a role in the initiation of biofilm formation, as deletion of the *nhbA* gene in a NalP^+^ strain that follows an eDNA-dependent strategy impairs biofilm formation (Arenas et al., 2013a).

The *autA* gene is present in the genomes of various *Neisseria* species. AutA is exposed at the cell surface where it binds eDNA and interacts with AutA on neighboring cells. Its synthesis induces autoaggregation (Arenas et al., 2015a), which has drastic consequences for biofilm architecture. Expression of the *autA* gene is prone to phase variation due to slipped-strand mispairing at a tetranucleotide repeat within the coding region. In many strains, however, the gene harbors a premature stop codon, an insertion or a deletion that disrupts gene expression even if the gene is in phase at the tetranucleotide repeat. Expression of *autB* is also prone to phase variation and only very few strains express the gene suggesting a negative selection pressure against its expression (Arenas et al., 2016). Also AutB presumably binds DNA.

Interactions between different neisserial strains in the nasopharynx have been poorly studied so far. Previously, it has been shown that a strain following an eDNA-independent strategy of biofilm formation was outcompeted *in vitro* by strains following the eDNA-dependent one (Lappann and Vogel, 2010), whilst two strains both using an eDNA-dependent strategy could coexist in a biofilm (Lappann et al., 2010; Lappann and Vogel, 2010). However, the mechanisms of such inter-strain competition and interactions and the implications on biofilm structure remain to be elucidated. It is noteworthy, in this respect, that *Neisseria* strains synthesize a variety of toxins to compete congeners, including the TpsA (Arenas et al., 2013b) and MafB (Arenas et al., 2015b; Jamet et al., 2015) proteins, but their potential role in dual-strain biofilms has never been studied. Here, we analyzed the interactions between strains in dual-strain biofilms and used different mutants to understand the mechanisms behind.

## Materials and methods

### Bacterial strains and growth conditions

Bacterial strains used in this study are listed in Table 1 and Table S1. Neisserial strains were grown overnight on GC agar base medium (OXOID) supplemented with Oxoid™ Vitox at 37°C in a CO_2_-enriched atmosphere provided by a candle jar. When appropriate, erythromycin (7 μg ml^−1^), kanamycin (100 μg ml^−1^), chloramphenicol (10 μg ml^−1^), rifampicin (50 μg ml^−1^) or gentamicin (60 μg ml^−1^) was added to the medium. BB-1 and HB-1 are capsule-deficient derivatives of strains B16B6 (Frasch and Chapman, 1972) and H44/76 (Holten, 1979), respectively, which were isolated from patients with meningococcal disease in the United States and Norway, respectively. Strain α14 was isolated in Germany from a healthy carrier (Claus et al., 2005). A rifampicin-resistant derivative of HB-1Δ*nalP* was selected after plating the strain on GC plates supplemented with rifampicin.

**Table 1:** **Characteristics of strains HB-1, BB-1, and α14**.

	**HB-1**	**BB-1**	**α14**
Lineage	cc32	cc11	cc53
Isolation	Invasive	Invasive	carrier
Capsule	−	−	−
Twitching motility	+	+	−
NalP	+	+	−
AutA	−^*^	−^*^	+
DNase sensitivity biofilms	+	−	+

* *No full-length AutA can be produced even if the gene is in phase at the tetranucleotide repeats because of the presence of a premature stop codon*.

For liquid cultures and biofilm experiments, bacteria grown on plates were resuspended in Tryptic Soy Broth (TSB) (Scharlau) or RPMI (Gibco) to an optical density at 550 nm (OD_550_) of 0.1 and incubated in polystyrene cell culture flasks with shaking (120 rpm) until they reached the exponential growth phase (OD_550_ of ~1.0). To induce the expression of genes from plasmids, 0.1 mM isopropyl-β-D-l-thiogalactopyranoside (IPTG) was added to the medium.

*Escherichia coli* strain DH5α was grown in lysogeny broth (LB) with shaking or on solid LB medium at 37°C. For plasmid selection, the following antibiotics were included in the medium: kanamycin (50 μg ml^−1^), chloramphenicol (25 μg ml^−1^) or gentamicin (20 μg ml^−1^).

### Plasmid construction

All plasmids and PCR primers used in this study are listed in Tables S1, S2, respectively. Regular PCR reactions were performed by using Dream Taq-DNA polymerase (Thermo Scientific), whilst PCR fragments generated for cloning were obtained using the High Fidelity polymerase (Roche Diagnostics GmbH) or Phusion DNA polymerase (Thermo Scientific). For purification of PCR products, the Wizard® SV Gel and PCR Clean-Up System (Promega) were used. Plasmids were isolated with the commercial E.Z.N.A.® Plasmid Mini Kit I (Omega Bio-Tek). PCR products and plasmids were both digested for 2 h with appropriate restriction enzymes (Thermo Scientific) for which cleavage sites were included in the primers, purified and ligated using T4 DNA ligase (5 U/μl) (Thermo Scientific).

For the preparation of pIN plasmids, plasmids pCRT_hrtA and phrtA_gm_rfp containing the *hrtA* region (Figure S1A) were used. The *gfp* gene together with an upstream region containing the *lac* promoter were amplified by PCR from plasmid mut3.1 and inserted into plasmid pCRT_hrtA via MluI and PpuMI digestion. Subsequently, a gentamicin-resistance cassette amplified by PCR from phrtA_gm_rfp was inserted via PpuMI, resulting in plasmid phrtA_gm_gfp. Fragments of different length (221–273 bp) located upstream of the *opaB* gene and containing the −35 and −10 boxes of the *opaB* promoter (*opaB*P) were amplified by PCR from *Ng* strain FA1090 using different primer pairs (Table S2) and used to generate three promoter variants: *opaB*P_M_, *opaB*P_L_, and *opaB*P_H_ (Figure S1C). The sequence of *opaB*P_M_ differs with respect to *opaB*P in four nucleotides creating an NheI site located between the ribosome-binding site (RBS) and the start codon (Figure S1C). This fragment and a *gfp* gene, amplified by PCR from plasmid phrtA_gm_gfp, were digested with NheI and ligated together. The resulting ligation product was purified and inserted into phrtA_gm_rfp via MluI and Van91I resulting in plasmid pIN_M_ (Figure S1B). The fragment amplified for *opaB*P_H_ was 18 nucleotides smaller than that for *opaB*P_M_, replacing 52 nucleotides immediately upstream of the start codon by 34 nucleotides. This fragment was inserted into phrtA_gm_gfp and phrtA_gm_rfp upstream of the gene that encodes the fluorescent protein via MluI and SmaI. The resulting plasmids, pIN_H_ and pIN_H−RED_, respectively, contain a different sequence between the -10 box and the start codon as compared with pIN_M_ (Figure S1C). The fragment amplified for *opaB*P_L_ was 3 nucleotides larger than *opaB*P, replacing 32 bp by 35 bp immediately upstream of the start codon, and this was cloned into phrtA_gm_gfp via MluI and SmaI. The resulting plasmid, pIN_L_, contains a similar sequence between the -10 box and start codon as pIN_H_ but contains 21 additional bp in *opaB*P (Figure S1C). The correct insertion of the fragments was confirmed by PCR and subsequent sequencing of plasmids at the Macrogen sequencing service (Amsterdam).

### Biofilm formation

Biofilms were formed under static conditions in 24-wells plates as previously described (Arenas et al., 2013a) with modifications. Briefly, 5-h old cultures in TSB were adjusted to an OD_550_ of 1, and 500-μl samples were seeded per well on a round glass cover slip. For mixed biofilms, 500-μl samples were mixed 1:1, unless mentioned otherwise, and the mixture was placed in the well. After 15 h of incubation, the medium was removed from each well, and the biofilm was washed twice with de-ionized water. Biofilms were chemically fixed with 0.1 mM PBS (137 mM NaCl, 2.7 mM KCl, 10 mM Na_2_HPO_4_, and 1.8 mM KH_2_PO_4_) containing 2% formaldehyde for 2 h for microscopy analysis. In some experiments, bacteria were harvested from cultures by centrifugation (4,500 g for 5 min) and resuspended in the supernatant recovered from the culture of another strain before initiation of biofilm formation. To determine DNase sensitivity of biofilm formation, cultures were treated with 100 mg ml^−1^ of DNase I and biofilm formation was determined using crystal violet as described (Arenas et al., 2013a).

### Microscopy, image analysis, and films

Fixed, 15-h old biofilms were used for microscopy. All microscopic observations and image acquisitions were performed using a Zeiss LSM 700 confocal laser scanning microscope (Carl Zeiss, Germany) equipped with detectors and filter sets for monitoring fluorescence. Images were obtained using a 20x/0.8 Plan-Apochromat, a 40x/1.30 Plan-Neofluar oil or a 63x/1.40 Plan-Apochromat oil objective. Phenotypes were considered when at least observed in three independent experiments performed in duplicate. For the analysis of the structural parameters of the biofilm (biomass, average thickness, roughness coefficient, and surface to volume ratio), image stacks at 0.4 μm z-intervals were acquired and analyzed with the program COMSTAT (Heydorn et al., 2000) in the image processing environment ImageJ (v1.48, NIH, http://imagej.nih.gov/ij/).

To determine the level of expression of fluorescent proteins expressed by each pIN variant, cell were fixed by adding formaldehyde (1% v/v) to an exponentially growing culture as described (Arenas et al., 2008) and formalin-fixed cells were visualized by fluorescence microscopy. An outline was drawn around 20 formalin-fixed cells, and the mean fluorescence was measured, along with several adjacent background readings. Then, the total corrected cellular fluorescence (TCCF) = integrated density – (area of selected cell × mean fluorescence of background readings), was calculated for each bacterium. Statistical analyses were performed considering TCCF values obtained from each construct.

To determine twitching motility in biofilms, time-lapse videos of fluorescent bacteria in 0.5- and 2-h old biofilms of strains following an eDNA-dependent and -independent strategy, respectively, were recorded. Sixty images were taken at 0.1-ms intervals. For the HB-1Δ*pilE* mutant, biofilms were stained with a LIVE/DEAD BacLight Bacterial Viability Kit (Molecular Probes).

### Preparation of samples, SDS-page and western blotting

Liquid cultures grown to an OD_550_ of ~2.0 were centrifuged (4,500 g for 5 min), and the resulting cell pellet was resuspended in H_2_O to an OD_550_ of 10. The spent media were centrifuged at 16,000 g for 15 min to remove residual cells, and proteins were precipitated from the supernatant with 10% (w/v) trichloroacetic acid in H_2_O. After centrifugation (16,000 g, 15 min), the resulting pellets were washed with ice-cold acetone, air-dried and resuspended in H_2_O. For analysis of protein production in biofilms, 15-h old biofilms were disrupted by mechanical forces, and bacterial cells were peletted (4,500 g for 5 min) and resuspended in H_2_O to a final OD_550_ of 10. For analysis of secreted proteins in biofilms, biofilms and planktonic cells were mixed by pipetting and the suspensions were centrifuged at 16,000 g for 15 min to remove cells and debris, and proteins were precipitated from the supernatants as described above. All samples were resuspended in double-strength sample buffer for electrophoresis and boiled for 10 min.

For SDS-PAGE, the Mini-PROTEAN® Electrophoresis System (Bio-Rad) was used. After electrophoresis, gels were stained with Coomassie Brilliant blue. Western blot analysis was performed as described (Arenas et al., 2015a). Blots were developed with SuperSignal® West Pico Chemiluminescent Substrate (Thermo Scientific) for 1 min at room temperature, and the image was acquired on a light-sensitive film (Fuji Medical X-Ray Film) or in a bio-imaging system (BioRad). The antiserum directed against GFP was purchased from Sigma-Aldrich. The antisera directed against the translocator domain of IgA protease (Roussel-Jazédé et al., 2014), the translocator domain of NalP (Oomen et al., 2004), and the TPS domain of TpsA of system 1 (van Ulsen et al., 2008) were previously described. The antiserum directed against MafA_MGI−3_ is from our laboratory collection, and the monoclonal antibody SM1 directed against PilE was generously provided by John Heckels (University of Southampton).

### Quantification of live bacteria in the biofilm

Bacteria with different antibiotic resistance markers were used to discriminate between two strains (indicated in Table S1). The biofilm was washed twice with Hanks' Balanced Salt Solution Gibco® (ThermoScientific). To disrupt the aggregates, biofilms were incubated for 15 min at room temperature with a solution containing 0.5 mg ml^−1^ of DNase I (Sigma-Aldrich) and 0.02 mg ml^−1^ of proteinase K (ThermoScientific) diluted in TSB. Complete disruption of aggregates was confirmed by microscopy. The bacterial suspensions were serially diluted and plated on GC agar containing appropriate antibiotics, and the colony-forming units (CFU) were quantified after 12 h of incubation.

### Statistical analysis

For statistical comparisons, data from at least three independent experiments performed in duplicate were considered. To determine the structural parameters of the biofilm, at least five image stacks of each sample were obtained from representative experiments. Data were analyzed using an unpaired statistical *t*-test with GRAPH PAD v 6.0 (Graph Pad Software, Inc.).

## Results

### Engineering fluorescent neisseriae

To facilitate the visualization of bacteria by fluorescence microscopy and the discrimination between different strains or species in mixed biofilms, we designed new plasmids to integrate genes encoding fluorescent proteins into the chromosome. A high rate of transformation (*hrtA)* region (Claus et al., 1998) present in the chromosome of several *Neisseria* species with >90% of sequence identity (Figure S1A) was exploited for this purpose. In a plasmid containing this region, we introduced (i) the promoter of the *opaB* gene (*opaB*P), which encodes an abundant protein expressed in some *Neisseria* species and previously used for protein production (Ramsey et al., 2012), (ii) a gene encoding green or red fluorescent protein (GFP or RFP, respectively), and (iii) a gentamicin-resistance cassette (Figure S1B). This plasmid was designated pIN (plasmid for Integration in *Neisseria*). Three different *opaB*P variants were tested in the pIN backbone (Figure S1C), and they were called *opaB*P_H_, *opaB*P_M_, and *opaB*P_L_. The pIN variants, which were called pIN_H_, pIN_M_, and pIN_L_, respectively, were used to transform *Nm* strain HB-1 selecting for gentamicin-resistant recombinants. To test protein expression levels, the intensity of fluorescence emission was acquired for individual bacteria and analyzed with the image processing program ImageJ. The results revealed large differences in fluorescence intensity between the promoter variants in the order *opaB*P_H_ > *opaB*P_M_ > *opaB*P_L_, in accordance with protein production levels detected on Western blots (Figure S1D). The fluorescence of pIN_H_-derived transformants was visualized in all our imaging devices and allowed for the discrimination of fluorescent strains in mixed biofilms. Therefore, this plasmid was used to generate fluorescent bacteria. Indeed, we could generate fluorescent bacteria in strains of *Nm, Nl*, and *Ng* transformed with this plasmid (Figure S1E).

### Biofilm structure of fluorescent neisseriae

**Figure 1:**
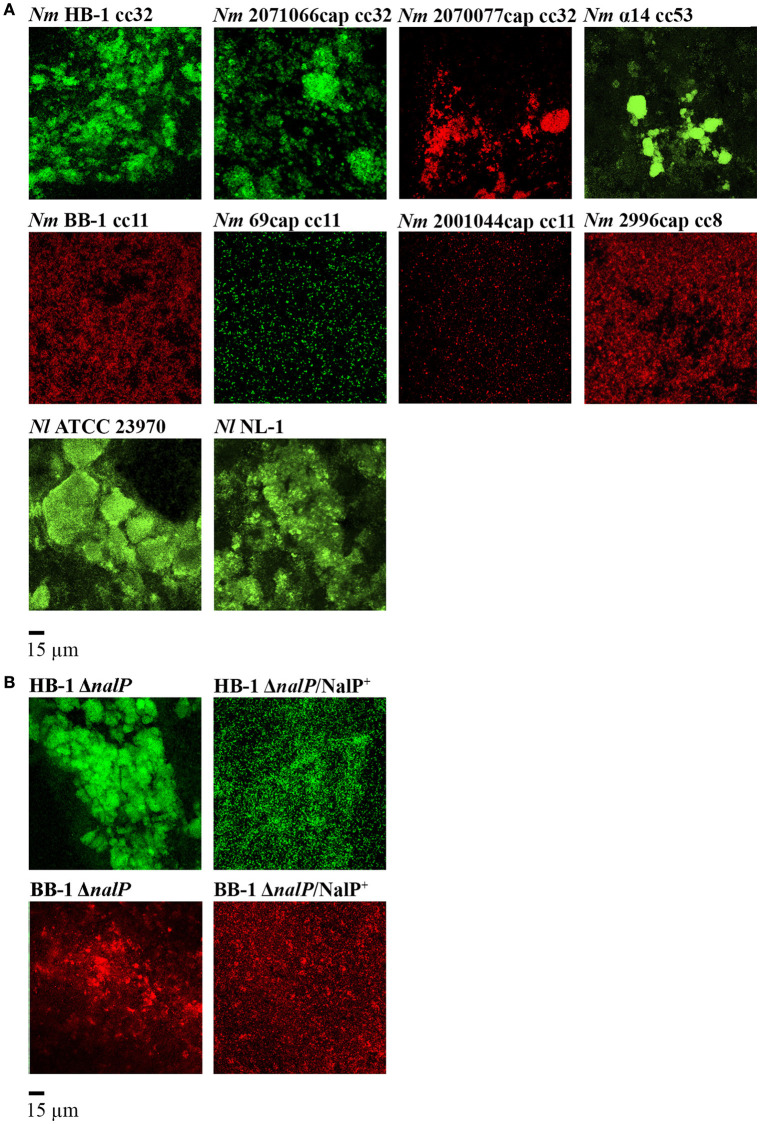
**Biofilm organization in various fluorescent ***Neisseria*** strains. (A)** Biofilms of various strains of *Nm* and *Nl*. The clonal complex (cc) of the *Nm* strains is indicated. **(B)** Influence of *nalP* inactivation on biofilm architecture. Wild-type phenotypes are shown in **(A)**. HB-1Δ*nalP/*NalP^+^ and BB-1Δ*nalP/*NalP^+^ represent *nalP* mutants overexpressing NalP from plasmid pEN300. All strains harbor an *opaB*P_H_-gfp or *opaB*P_H_-rfp inserted into *hrtA* locus. Representative pictures from at least four experiments with two technical replicates are shown.

To study the biofilm structure of various *Nm* and *Nl* strains, biofilms of fluorescent bacteria were formed on glass and visualized by confocal microscopy. All strains used were capsule deficient, as capsule has been reported to inhibit biofilm formation on abiotic surfaces (Yi et al., 2004; Lappann et al., 2006). Figure 1A shows the structures of 15-h old biofilms of various strains. Biofilms consisted of cell clusters, but the size, dispersion and number of the clusters differed largely between both species and between strains of the same species. *Nm* strains generally formed smaller clusters than did the *Nl* strains. Also, *Nm* strains of cc8 and cc11 formed much smaller and less compact clusters with, together, a larger coverage of the substratum than strains of cc32 and cc53 (Figure 1A). It is noteworthy that strains of cc8 and cc11 use an eDNA-independent strategy of biofilm formation in contrast to strains of other cc (Lappann et al., 2010). Both *Nl* strains formed biofilms that were sensitive to DNase I (data not shown). Thus, these results confirmed that aggregation is a common feature during neisserial biofilm formation.

### eDNA-binding proteins contribute to cell aggregation in single-strain biofilms

AutA and type IV pili are known to be involved in bacterial aggregation during biofilm formation (Lappann et al., 2006; Arenas et al., 2015a). Here, we explored the contribution in this process of eDNA-binding proteins, which are cleaved from the cell surface by the protease NalP. Figure 1B shows the biofilm structure of mutants of *Nm* strains HB-1 and BB-1 lacking *nalP*. The *nalP* mutants formed bigger and more compact microcolonies than the corresponding wild types (Figure 1A), although the difference was less pronounced in BB-1, in accordance with the eDNA-independent strategy of biofilm formation of this strain. The stronger aggregation of HB-1Δ*nalP* is not due to increased piliation, as Western blotting assays showed a similar production of PilE, the major pilus subunit, in the wild type and the *nalP* mutant (Figure S2A). Since cc11 strain BB-1 produces a different type of pilin that is not recognized by the antibodies (Cehovin et al., 2010) we could not verify *pilE* expression in this strain and its *nalP* mutant. The *autA* gene is disrupted in HB-1 and BB-1 because of the presence of a premature stop codon (Arenas et al., 2015a) and can, therefore, not play a role in the differences between the NalP-producing and non-producing strains. Furthermore, aggregation was severely reduced when NalP was expressed *in trans* from plasmid pEN300 in the *nalP* mutants (Figure 1B) demonstrating that the increased aggregation of the *nalP* mutants is a direct effect of the lack of NalP synthesis. Microscopic examination of log-phase precultures grown under shaking conditions showed the presence of only few very small aggregates in the *nalP* mutant of HB-1 but not in the wild type (Figure S2B). The size of these aggregates does not match those observed in biofilms (Figure S2B). Probably, the abundance of eDNA at the surface of the *nalP* mutant cells facilitates aggregation and thereby microcolony formation. Such interactions may occur already in liquid cultures, but they are disrupted by physical forces during shaking. In conclusion, these results show that microcolony formation occurs during biofilm formation and that *nalP* expression influences this process, presumably by cleaving eDNA-binding proteins from the cell surface. Thus, these data expand the previously established role of NalP during the initiation of biofilm formation by demonstrating its effect on biofilm structuring.

### Interbacterial interactions in dual-strain biofilms

The high rates of *Nm* colonization (Sim et al., 2000) suggest an intense traffic of strains within the nasopharynx. Consequently, strains could interact with each other to compete or to benefit during colonization. To study how different strains affect each other during biofilm formation, we analyzed pairwise combinations of three *Nm* strains in biofilm experiments. We selected strains HB-1, BB-1, and α14 because different traits relevant for biofilm formation. HB-1 and BB-1 were chosen as representatives of strains following an eDNA-dependent and -independent strategy of biofilm formation, respectively (Arenas et al., 2013a). Strain α14 was chosen because it produces AutA, which causes autoaggregation and thereby affects biofilm architecture (Arenas et al., 2015a). HB-1 and BB-1 do not produce AutA (Arenas et al., 2015a). Other relevant characteristics of these strains are listed in Table 1.

First, several relevant properties of these strains were further analyzed. HB-1 and BB-1 clearly showed twitching motility in biofilms (Videos 1, 2, respectively, in Supplementary Material). Strain α14, however, showed no twitching motility, similar as a *pilE* mutant of HB-1 (Videos 3, 4, respectively, in Supplementary Material). Interestingly, Western blot analysis revealed a considerable difference in the electrophoretic mobility of the PilE proteins of HB-1 and α14 (Figure S2A), even though the sequences of these proteins are identical according to the available genome sequences (Schoen et al., 2008; Piet et al., 2011). Additionally, the genome sequence of α14 showed that *nalP* is out of phase; Western blotting confirmed that NalP is not synthesized in this strain (Figure S2A). All relevant properties of the three strains are listed in Table 1.

**Figure 2:**
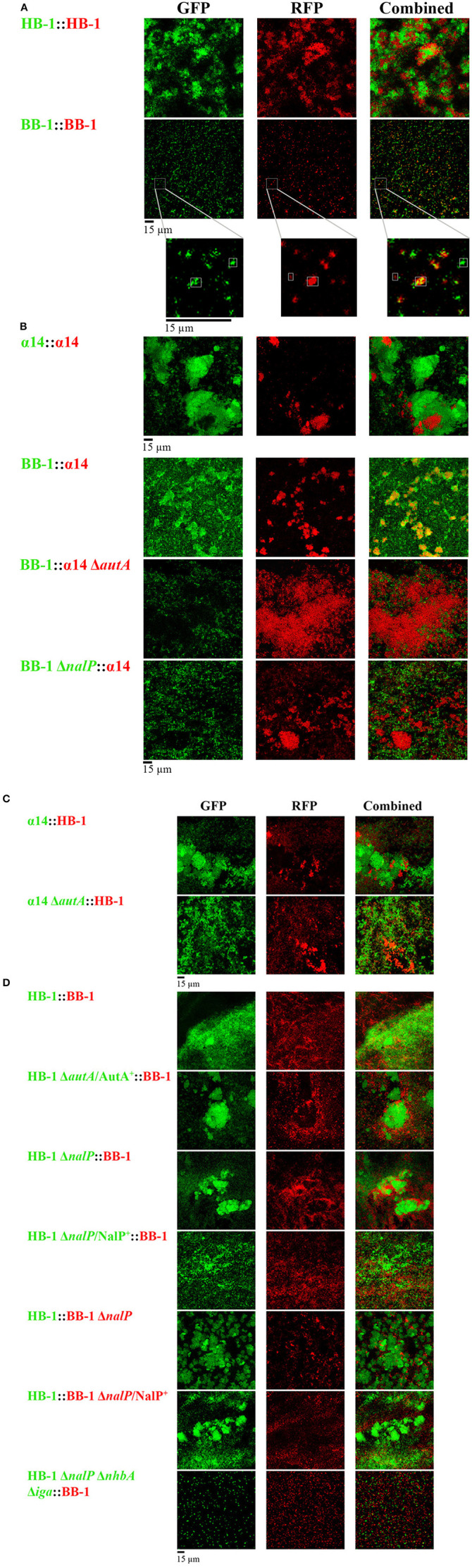
**Interbacterial interactions in dual-strain biofilms. (A)** Biofilms constituted of two differently labeled derivatives of strain HB-1, α14 or BB-1. In the enlargements of the BB-1 biofilms, the positions of three clusters are highlighted, one consisting exclusively of GFP-labeled cells, one consisting exclusively of RFP-labeled cells, and one intermixed cluster consisting of both GFP- and RFP-labeled cells. **(B)** Combinations of α14 and BB-1 and several mutant derivatives. **(C)** Combinations of α14 or its *autA* mutant derivative with HB-1. **(D)** Combinations of HB-1 and BB-1 and several mutant derivatives. Strain names at the left are shown in green or red, reflecting the expression of GFP or RFP, respectively. Designations AutA^+^ and NalP^+^ indicate expression of AutA and NalP from plasmids pFPAutA and pEN300, respectively. For clarity, strain HB-3Δ*nhbA*Δ*iga* (Table S1) is indicated here as HB-1Δ*nalP*Δ*nhbA*Δ*iga*. However, in contrast to strain HB-1Δ*nalP*, the isogenic strain HB-3 contains markerless deletions of the *nalP* gene and the capsule locus, which was necessary to combine multiple mutations into a single strain (Arenas et al., 2013a). Individual and combined fluorescence is displayed.

Each strain was grown independently in TSB, and, after adjusting them to the same OD, they were mixed 1:1 for biofilm formation. First, green- and red-fluorescent variants of strain HB-1 were combined (Figure 2A). Both variants of the strain formed separate clusters with hardly any intermixing, although these separate clusters extensively interacted (Figure 2A). A similar result was observed when green- and red-fluorescent variants of α14 were mixed (Figure 2A). Presumably, these separate clusters result from clonal outgrowth. In contrast, small intermixed clusters were abundantly found when green- and red-fluorescent variants of BB-1 were combined (Figure 2A), suggesting that the less aggregative nature of this strain allows for intermixing.

Next, strains of different genetic backgrounds were mixed. Interestingly, strain BB-1, which forms only very small clusters dispersed over the substratum in single-strain biofilms (Figure 1A), formed large clusters, which coincided with those of α14, in dual-strain biofilms (Figure 2B), indicating that these strains can form intermixed microcolonies. In contrast, HB-1 and α14 formed separate but interacting clusters (Figure 2C), whilst strains BB-1 and HB-1 attached to the substratum without much apparent association between the clusters formed (Figure 2D). Lack of intermixing without much association between clusters was also observed in most cases when *Nm* strains were combined with *Nl* strains (Figure S3). Only associations of clusters of α14 with those of the *Nl* strains were observed.

Next, we studied the possible role of AutA in the formation of intermixed clusters of α14 and BB-1. Indeed, intermixing was drastically reduced when an *autA* mutant of α14 was used, and separate but interacting clusters of the two strains were observed (Figure 2B). Unfortunately, the introduction of plasmid pFPAutA in the *autA* mutant of α14 for complementation studies failed for unknown reasons (Arenas et al., 2015a). To further investigate the possible role of AutA in the formation of intermixed clusters with BB-1, the AutA-encoding plasmid was introduced in another genetic background, i.e., HB-1. The synthesis of AutA in HB-1, which was confirmed on Western blots (Figure S4A), increased the interaction of its clusters with those of BB-1, but it did not lead to intermixed clusters (Figure 2D). Thus, additional traits of α14, such as the absence of NalP or of twitching motility (Table 1), may contribute to its ability to form intermixed clusters with BB-1.

Considering that NalP inactivation increased aggregation in single-strain biofilms (Figure 1B), we asked whether the presence or absence of NalP may also affect the interaction between different strains in mixed biofilms. Combinations of strains HB-1 and BB-1 with their respective *nalP* mutant derivatives generated biofilms consisting of separate clusters of the individual strains, which, however, extensively interacted with those of the other strain as evidenced by their co-localization on the substratum (Figure 2D). The ectopic expression of *nalP* from pEN300 in both *nalP* mutants drastically reduced these interactions (Figure 2D). Furthermore, the combination of BB-1 with a derivative of HB-1 lacking, besides *nalP*, also the *nhbA* and *iga* genes showed only small clusters of the HB-1 derivative that did not interact with those of BB-1 (Figure 2D), confirming that the increased aggregation observed for the *nalP* mutant is mediated by the increased cell-surface exposure of intact NHBA and αP in this strain. Interestingly, inactivation of *nalP* in strain BB-1 interfered with the formation of intermixed microcolonies with α14 and led to the formation of separate clusters of the individual strains, which barely interacted (Figure 2B). Probably, in this case, the increased interaction between the BB-1 cells due to the inactivation of NalP prevents intermixing with the α14 cells. In conclusion, *Nm* strains can form microcolonies constituted of mixed lineages or separate clusters of individual strains that variably interact depending on the strains studied and on the synthesis of AutA and/or NalP.

### Characterization of single- and dual-strain biofilms

**Figure 3:**
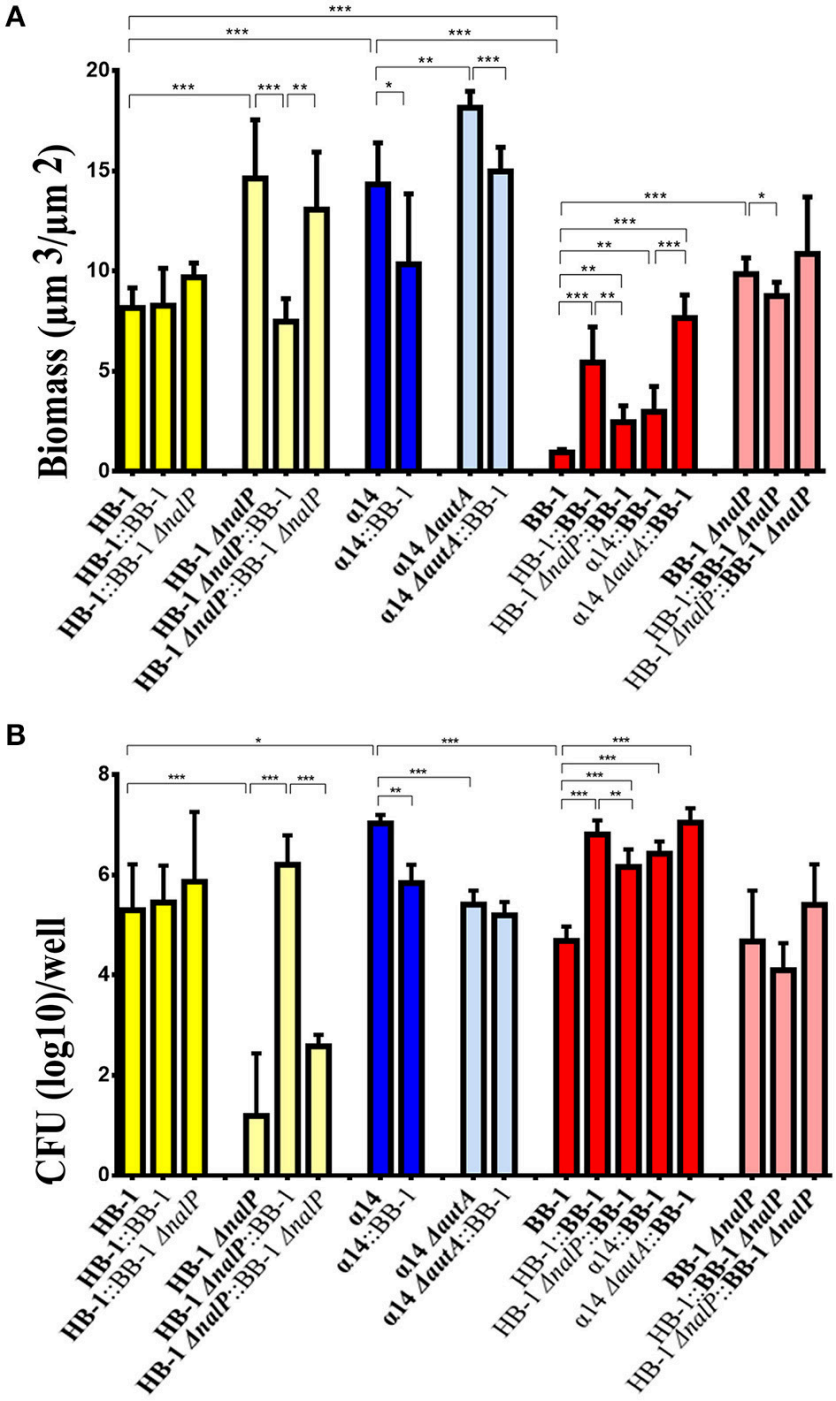
**Characteristics of single- and dual-strain biofilms**. Biofilms were formed of various combinations of *Nm* strains HB-1 and BB-1 and their *nalP* mutant derivatives, and α14 and its *autA* mutant derivative and biomass and CFU were determined. The strains present in the consortium are shown at the bottom of each panel. The data shown are for the strain indicated in bold. **(A)** The biomass of biofilms was calculated for each strain using COMSTAT. The biomass for each strain in the consortium is shown separately. **(B)** Numbers of CFU. The CFU for each strain in the consortium is shown. To allow for strain discrimination in dual-strain biofilms, strains with different antibiotic-resistance markers were used as indicated in Table S1. The CFU for each strain were determined by plating on GC medium containing kanamycin, rifampicin or gentamicin and overnight incubation. Results are means and standard deviations of three independent experiments. Statistically significant differences are marked with one (*P* < 0.05), two (*P* < 0.005) or three asterisks (*P* < 0.0005) (unpaired *t*-test).

To gain more insight into the implication of AutA and NalP synthesis on biofilm architecture, the individual contribution of each strain in mixed biofilms was analyzed with COMSTAT software and statistically compared with results of single-strain biofilms. Single-strain biofilms of HB-1, BB-1, and α14 differed considerably; the biomass, that reflects the amount of both live and dead bacteria in the biofilms, was in the order α14 > HB-1 > BB-1 (Figure 3A). Also other biofilm parameters, such as thickness, surface/volume ratio, and roughness, differed (Figure S5). To test bacterial viability, the biofilms were disrupted and the numbers of CFU were determined on selective GC plates. Viability was determined at 15, 24, and 48 h after initiation of biofilm formation and found to decrease drastically for HB-1 and α14 at 48 h (data not shown). To better compare the differences in viability between wild types and mutants of the three strains, results are shown for 24-h old biofilms (Figure 3B), although similar results were observed for 15-h old biofilms. In single-strain biofilms, the numbers of CFU of strains HB-1 and BB-1 did not differ but they were significantly different to those of α14 (Figures 3B).

Single-strain biofilms of the *nalP* mutants of BB-1 and HB-1 revealed higher biomasses compared to the corresponding wild types, but the numbers of CFU were similar or drastically decreased, respectively (Figures 3A,B). We did not find differences in viability between the wild types and the *nalP* mutants in liquid cultures (data not shown). The discrepancy between biomass and numbers of CFU indicates the presence of more dead cells in the biofilms of the *nalP* mutants and could be explained by the more compact clusters formed in the absence of NalP (Figure 1), which could result in lower exposure of the biomass to the nutrients. In summary, the pronounced aggregation observed in *nalP* mutants (Figure 1B) correlates with increased biomass and altered biofilm architecture but reduced bacterial viability.

In dual-strain biofilms, the biofilm biomass and structure of HB-1 remained almost unaltered when in consortium with BB-1 or BB-1Δ*nalP* (Figure 3A and Figure S5 for additional biofilm details). In contrast, the phenotypes of the HB-1Δ*nalP* mutant, such as increased biomass and reduced viability in biofilms, appeared to be complemented when this strain was grown with BB-1, but barely with BB-1Δ*nalP* (Figures 3A,B and Figure S5). This complementation was not caused by the cleavage of the αP from the cell surface of HB-1Δ*nalP* by NalP secreted by BB-1 as revealed by Western blotting (Figure S4B). Consistently, the phenotype of the HB-1Δ*nalP* mutant was not complemented when biofilms were formed in culture supernatants from BB-1. Apparently, the interaction of BB-1 with clusters of HB-1Δ*nalP* (Figure 2D) had an impact on the architecture of HB-1Δ*nalP* biofilms (Figure 3A and Figure S5) and on the survival of the HB-1Δ*nalP* bacteria within these biofilms (Figure 3B).

The biomass and viability of BB-1 in biofilms increased significantly when it was grown in a consortium with HB-1 or HB-1Δ*nalP* (Figures 3A,B). This shows that BB-1 benefits when mixed with HB-1, but BB-1 benefited less in consortium with HB-1Δ*nalP* than with HB-1. The biomass and viability of BB-1Δ*nalP* were barely affected when in consortium with HB-1 or HB-1Δ*nalP* (Figures 3A,B).

Biofilms of strain α14 had a lower biomass than those of its *autA* mutant derivative (Figure 3A). However, the number of CFU of the wild type in biofilms appeared higher (Figure 3B). In consortium with BB-1, the biomass and number of CFU of α14 and its Δ*autA* mutant derivative were similar or decreased (Figures 3A,B). The biofilm biomass and CFU of BB-1 were increased in consortium with α14 or α14Δ*autA* (Figure 3A) and also other biofilm parameters were altered (Figure S5). Together with the results of BB-1 grown in consortium with HB-1 or its *nalP* mutant derivative, these data indicate that biofilm formation of BB-1 increases when it is grown in consortium with other strains, and the degree of the increase is influenced by the synthesis of NalP or AutA in the partner strain.

### Interbacterial interactions under growth limiting conditions

Although *Nm* strains synthesize a variety of polymorphic toxins against congeners (Arenas et al., 2013b, 2015b; Jamet et al., 2015), the results in Figure 3B do not provide evidence of inter-bacterial competition in dual-strain biofilms. The expression of toxins in the biofilms was therefore evaluated in Western blotting assays. TpsA1 was detected in considerably higher amounts in the medium of disrupted biofilms than in liquid cultures grown under shaking conditions (Figure S4C). Large differences in the amounts of TpsA1 between the strains were detected in the order BB-1 > HB-1 > α14 (Figure S4C). As we have no suitable antisera available directed against MafB proteins, we had recourse to an antiserum directed against the MafA encoded by the Maf Genomic Island (MGI) 3, where the corresponding gene is located in an operon with a *mafB* gene (Jamet et al., 2015). This antiserum detected the MafA_MGI−3_ protein in biofilms of BB-1 and α14 (Figure S4D), indicating that also MafB_MGI−3_ is expressed in biofilms of these strains. MafA_MGI−3_ was not detected in HB-1, where the gene is disrupted by a premature stop codon. Notably, MafA_MGI−3_ was produced in higher abundance in biofilms than in liquid cultures (Figure S4D). In conclusion, the apparent absence of competition in the biofilm assays (Figure 3B) cannot be explained by the lack of expression of TpsA or MafB proteins.

**Figure 4:**
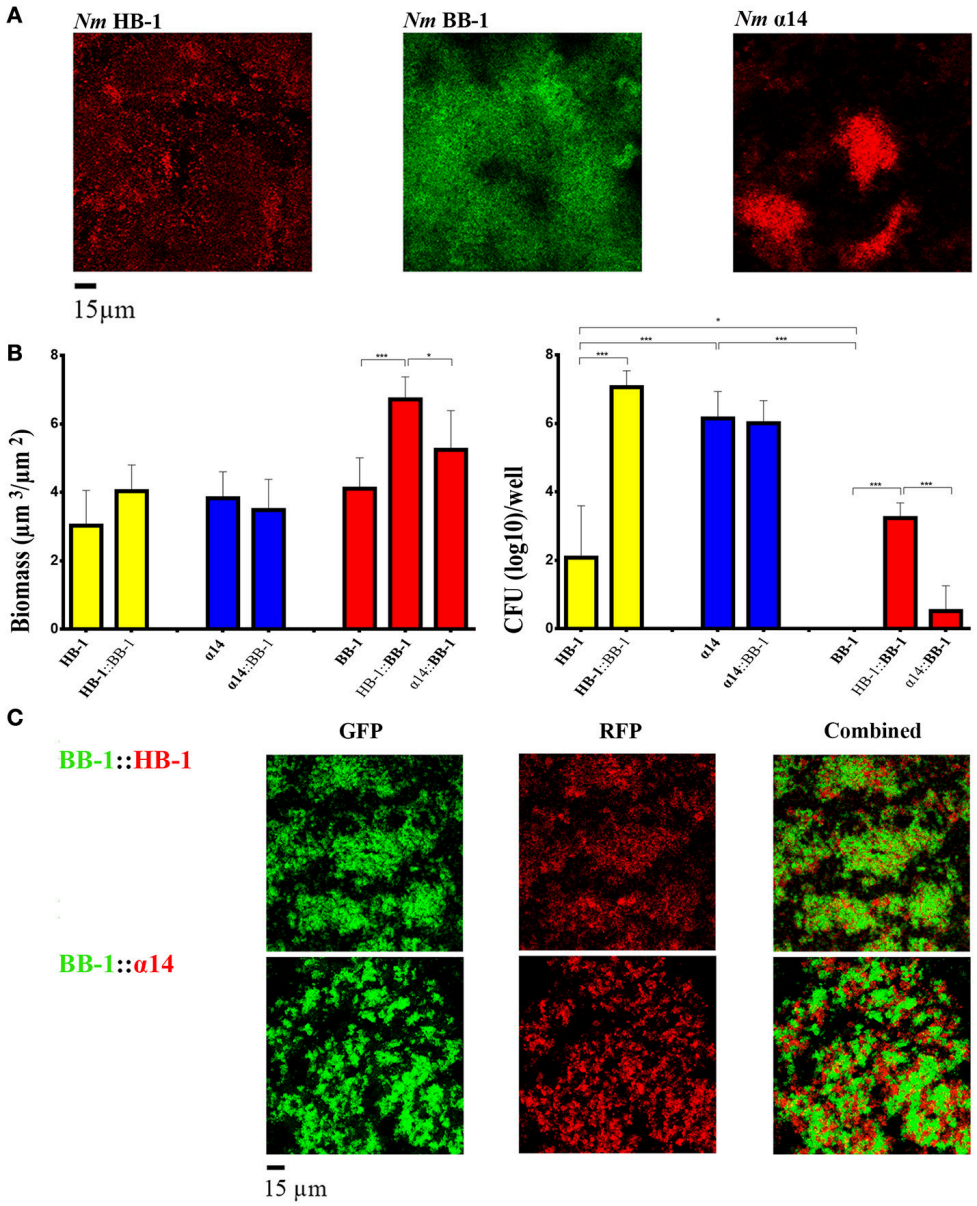
**Biofilm formation under nutrient limitation. (A)** Biofilms of various strains of *Nm* grown on RPMI medium. **(B)** Biomass (left panel) and CFU (right panel) in biofilms were calculated for each strain in single- and dual-strain biofilms as in Figure 3. The strains present in the consortium are shown at the bottom of each panel. The data shown are for the strain indicated in bold. Statistically significant differences are marked with one asterisk (*P* < 0.05) or with three asterisks (*P* < 0.0005) (unpaired *t*-test). **(C)** Interbacterial interactions in dual-strain biofilms constituted of BB-1 and HB-1 or α14.

Changing the ratio of HB-1 and BB-1 in dual-strain biofilms to 1:0.2 or 0.2:1, did not result in the suppression of the growth of either strain in the consortium (data not shown). Considering that competition between strains is particularly important under nutrient-limiting conditions, we also studied biofilm formation in RPMI medium, a synthetic medium that contains a low concentration of nutrient metals (Stork et al., 2010). In single-strain biofilms, the autoaggregative character of HB-1 and α14 already observed in TSB medium (Figure 1A) was retained (Figure 4A), while the biofilm biomass was reduced ~3-fold (compare Figure 3A and Figure 4B). Biofilms of BB-1 were dispersed on the substratum and showed less compact clusters as compared with those formed in TSB medium. Remarkably, the biofilm biomass of BB-1 in the nutrient-limited RPMI medium was increased ~4-fold relative to that in TSB (compare Figure 3A and Figure 4B). The three strains revealed a similar biomass in single-strain biofilms (Figure 4B), but the number of CFU in 24-h old biofilms varied by orders of magnitude in order α14 > HB-1 > BB-1 (Figure 4B). In dual strain biofilms, BB-1 formed compact clusters that interacted with those of α14 or HB-1 (Figure 4C). The biofilm biomass and CFU of BB-1 increased when in combination with HB-1 and, to a lesser extent, with α14 (Figure 4B). The number of CFU of HB-1 increased significantly in consortium with BB-1, whilst the CFU of α14 remained unaltered. In conclusion, under low-nutrient conditions, the biofilm architecture of the strains and the interactions with other strains may deviate from those in TSB, but we did not find evidence for competition.

## Discussion

What happens when two *Nm* strains colonize the nasopharynx simultaneously? Do they generate mixed biofilms, do they cooperate, or do they compete? Under conditions that are limiting for resources, such as in the nasopharynx, strong competition between *Neisseria* strains could be expected. This supposition is based on different studies. First, both *Nm* and *Nl* contain an arsenal of biological weapons, i.e., a variety of secreted toxins of the TpsA and MafB families (Arenas et al., 2013b, 2015b; Jamet et al., 2015). These toxins target congeners to inhibit their growth. Up to date, the growth-inhibitory activity of these systems has not been demonstrated in mixed biofilms, although *tpsA* gene expression was reported to be up-regulated in 48-h old biofilms (Neil and Apicella, 2009). Second, although colonization of the throat by different strains has been demonstrated in carriage studies, it was very rare and detected in only ~1% of the carriers (Caugant et al., 2007). Third, a recent study showed that a *Nm* strain that uses an eDNA-independent strategy for biofilm formation was outcompeted in a mixed biofilm by a strain following an eDNA-dependent strategy (Lappann et al., 2010). In our assays, the coexistence of two strains resulted in mutual or single benefits in some combinations. This was obvious for strain BB-1, which uses an eDNA-independent strategy of biofilm formation. When BB-1 was in consortium with strains HB-1 or α14 or their derivatives, which follow an eDNA-dependent strategy, this strain benefited as evidenced by increased biofilm biomass and CFU as compared to single-strain biofilms (Figures 3A,B). We also detected an increase in the biomass of biofilms under nutrient-limiting conditions when BB-1 was combined with HB-1 (Figure 4B). These results indicate that BB-1 may acquire a higher capacity to colonize the host when in consortium with a strain following an eDNA-dependent strategy for biofilm formation. Strains following the eDNA-independent strategy have a reduced capacity to initiate biofilm formation. They are often isolated from patients with meningococcal disease rather than from carriers (Lappann et al., 2010), a phenotype referred to as “spreaders” (Lappann and Vogel, 2010). In contrast, strains following an eDNA-dependent strategy have a high biofilm-forming ability; they are more often isolated from carriers than spreaders and are called “settlers” (Lappann and Vogel, 2010). Possibly, BB-1 uses the matrix dispersed on the substratum by eDNA-dependent strains to generate biofilms. In addition, BB-1 strongly associated with the clusters of NalP^−^ or AutA^+^ strains, even forming intermixed microcolonies with the AutA^+^ strain α14. We presume that in this case the eDNA-binding proteins (NHBA, αP or AutA) of the partner strains anchor large quantities of eDNA and subsequently other matrix components at their surface (Arenas and Tommassen, 2017), thereby promoting the interaction with cells and microcolonies of the same and other lineages. This would also explain the pronounced aggregation of Δ*nalP* mutants observed in single-strain biofilms (Figure 1B) as result of extensive associations of microcolonies. In any case, it appears that the capacity of BB-1 to colonize a host could be enhanced by public goods produced by other *Nm* strains. Another example of profiting from being in a consortium was strain HB-1Δ*nalP*, whose survival in mature biofilms increased by several orders of magnitude when in consortium with BB-1 (Figure 3B). A possible explanation for this effect is that BB-1, by interacting with microcolonies of HB-1Δ*nalP*, limits the aggregation of these microcolonies, whilst extensive aggregation may limit nutrient supply. Thus, these examples demonstrate that *Nm* strains can profit from growing in dual-strain biofilms.

Contrary to our expectations, we did not find evidence for strong competition in dual-strain biofilms even when we explored biofilm formation under nutrient limitation. Neisserial strains can coexist in a biofilm, but the level of interaction between strains is apparently different. In most cases, we observed biofilms constituted of separate clusters of the participating strains rather than of intermixed clusters. Such segregation was even observed between two differently labeled derivatives of strains HB-1 and α14 (Figure 2A), suggesting that these clusters were generated by clonal expansion of initially attached bacteria without recruitment and incorporation of new planktonic bacteria. This mechanism of microcolony expansion was also observed *in vitro* or in animal models for other mucosal pathogens, for example *Vibrio cholerae* (Nadell and Bassler, 2011; Millet et al., 2014). This spatial distribution avoids competition by contact-dependent growth-inhibition systems between lineages (Nadell et al., 2016) and explains the coexistence of microcolonies of different lineages within the same biofilm. Our results are consistent with those of Lappann and Vogel (2010) who reported that two *Nm* strains following the eDNA-dependent strategy of biofilm formation form mixed biofilms composed of separate clonal clusters. However, in contrast with our results, these authors reported that two differently labeled derivatives of the same strain completely intermix (Lappann et al., 2006), whilst a strain following an eDNA-independent strategy of biofilm formation was outcompeted by a strain following the eDNA-dependent one (Lappann et al., 2010; Lappann and Vogel, 2010). Differences in mixing behavior may be due to differences in pilus-mediated motility (Lappann et al., 2006; Oldewurtel et al., 2015). In our assays, HB-1 and BB-1 showed twitching motility in biofilms but α14 did not. The amino-acid sequences of PilE of α14 and HB-1 are identical, but PilE of α14 showed a lower electrophoretic mobility on gels as compared to that of HB-1 (Figure S2A), which could be explained by differences in posttranslational modifications. In any case, HB-1 and BB-1 were motile, and, whereas differently labeled BB-1 derivatives did form intermixed clusters, those of HB-1 did not. Possibly, the strongly aggregative nature of HB-1 prevents intermixing. The lack of inter-strain growth inhibition in our experiments could not be attributed to a deficiency in the production of toxins involved in inter-strain competition, i.e., TpsA and Maf family proteins (Figures S4C,D). Although we did not test all proteins involved in contact-dependent growth inhibition, those proteins tested were produced even in higher levels in biofilms as compared to planktonic cells in accordance with previous reports (Neil and Apicella, 2009). Thus, discrepancies with a previous study (Lappann and Vogel, 2010) could be attributed to the use of different strains or methodologies.

What is the role of the large variety of growth-inhibition systems in *Nm* if competition appears very limited? Interestingly, genetically identical *Burkholderia thailandensis* strains expressing different TpsA proteins formed clonal patches in biofilms, indicating a contribution of TpsA in microcolony segregation (Anderson et al., 2014). However, growth-inhibition systems in *Nm* do not seem to have a similar role in *Nm* as differently labeled lineages of the same strain expressing the same growth-inhibition systems already formed clonal patches in biofilms of HB-1 and α-14. Contact-dependent growth-inhibition systems were also proposed to have additional functions in self-recognition, interaction, and interbacterial signaling. TpsA contributes to biofilm development and structuration in *Nm* and other bacteria at least in part by mediating cell-cell interactions (Neil and Apicella, 2009; Anderson et al., 2012; Ruhe et al., 2015). Furthermore, transmission of the toxic domain of TpsA to immune kin cells has been shown to affect gene expression and promote biofilm formation and other social behaviors (Garcia et al., 2016). Possibly, these additional functions are more important than the growth-inhibitory properties of the systems.

Besides interactions between *Nm* strains, we observed that *Nm* coexist and interact with *Nl* in biofilms. *Nl* form part of the microbiome in the nasopharynx and colonize humans early in life. To the best of our knowledge, the formation of *Nl* biofilms has not been reported before. Probably, *Nl* uses similar mechanisms as *Nm*. *Nl* biofilms were sensitive to DNase I, and inspection of available genome sequences shows that also this species carries *nhbA, autA*, and *nalP* genes. However, it does not contain a gene for IgA protease. Thus, one may speculate that the exposition of eDNA-binding region of unprocessed NHBA at the cell surface explains the aggregative phenotype of these strains and facilitates their interaction.

To our knowledge, this is the first study that reports cooperation between *Nm* strains in biofilms. Cooperative behaviors are found in multispecies biofilms in nature and they can be relevant in host-pathogen interactions (Røder et al., 2016). For example, bacterial coaggregation is an important cooperative interaction between oral bacteria of different species, which facilitates the co-adhesion of bacteria to the tooth surface (Rickard et al., 2003). Our results point in this direction. We observed that a consortium of two strains can provide mutual or single benefits and this may facilitate colonization. The variable production of AutA and NalP proteins balances the spatial distribution of the strains in the biofilm, as well as the biomass and final architecture of the biofilm, which may be key to protection against mechanical forces derived from mucus flow, coughing or swallowing. Possibly, the long term capacity of each microcolony to resist the host's defenses explains the rare coexistence of two *Nm* strains in one host.

## Author contributions

JA conceived the study; AR, JP, JT, and JA designed the experiments; AR, JP, ER, and JA performed the experiments; AR, JP, JT, and JA analyzed the data and AR, JT, and JA wrote the paper. All authors have read and approved the manuscript.

### Conflict of interest statement

The authors declare that the research was conducted in the absence of any commercial or financial relationships that could be construed as a potential conflict of interest.

## Supplementary material

Video 1
https://figshare.com/s/f8826227b634bb63512a

Video 2
https://figshare.com/s/d05f4d8add7e3957d0c2

Video 3
https://figshare.com/s/2aa04f555f793c10d646

Video 4
https://figshare.com/s/eb1abba727476892fc55
