# Supplementary material for: Interstrain Cooperation in Meningococcal Biofilms: Role of Autotransporters NalP and AutA
Source: Front Microbiol. 2017 Mar 22;8:434. doi: 10.3389/fmicb.2017.00434 (PMC5360712; doi:10.3389/fmicb.2017.00434)
Supplement: Supplementary file 1 [file Table1.pdf]

TABLE S1 | Bacterial strains and plasmids used in this study

Strain or plasmid	Description	Source/Reference
**Strains**[a]		
E. coli		
DH5α	Cloning strain	UU
DH5α_GFP	DH5α carrying plasmid pIN$_{H.}$ Gm^R, Amp^R, Kan^R	This study
N. lactamica		
NL-1		UU
NL-1_GFP	NL-1 with insertion of *opaB*P$_H$-gfp into *hrtA* locus. Gm^R	This study
ATCC 23970	Reference strain	UU
23970_GFP	ATCC 23970 with insertion of *opaB*P$_H$-gfp into *hrtA* locus. Gm^R	This study
N. gonorrhoeae		
FA1090	Reference strain	UU
FA1090_GFP	FA1090 with insertion of *opaB*P$_H$-gfp into *hrtA* locus. Gm^R	This study
N. meningitidis		
2996cap	Derivative of 2996; cc8; capsule locus replaced by *ery*. Ery^R	[1]
2996cap_RFP	2996cap with insertion of *opaB*P$_H$-rfp into *hrtA* locus. Ery^R, Gm^R	This study
α14	Strain isolated from carrier; cc53	[2]
α14_GFP (e)	α14 with insertion of *opaB*P$_H$-gfp into *hrtA* locus. Gm^R	This study
α14 Δ*autA* (f)	α14; *autA* replaced by *kan.* Kan^R	[3]
α14 Δ*autA*_GFP	α14 Δ*autA* with insertion of *opaB*P$_H$-gfp into *hrtA* locus. Gm^R, Kan^R	This study
α14_RFP	α14 with insertion of *opaB*P$_H$-rfp into *hrtA* locus. Gm^R	This study
α14 Δ*autA*_RFP	α14 Δ*autA* with insertion of *opaB*P$_H$-rfp into *hrtA* locus. Gm^R, Kan^R	This study
BB-1	B16B6; capsule locus replaced by *ery*; cc11. Ery^R	[1]
BB-1^{Rif} (a) (c) (e) (f)	Rifampicin-resistant derivative of BB-1. Ery^R, Rif^R	[4]
BB-1_RFP	BB-1 with insertion of *opaB*P$_H$-rfp into *hrtA* locus. Ery^R, Gm^R	This study
BB-1 Δ*nalP* (b)	BB-1; *nalP* replaced by *kan.* Ery^R, Kan^R	[1]
BB-1 Δ*nalP*_RFP (d)	BB-1 Δ*nalP* with insertion of *opaB*P$_H$-rfp into *hrtA* locus. Ery^R, Gm^R, Kan^R	This study
BB-1 Δ*nalP*/NalP$^+$	BB-1; *nalP* replaced by *kan* and carrying pEN300. Ery^R, Cam^R, Kan^R	UU
BB-1 Δ*nalP*/NalP$^+$_RFP	BB-1 Δ*nalP*/NalP$^+$ with insertion of *opaB*P$_H$-rfp into *hrtA* locus. Ery^R, Cam^R, Kan^R, Gm^R	This study
BB-1_GFP	BB-1 with insertion of *opaB*P$_H$-gfp into *hrtA* locus. Ery^R, Gm^R	This study
BB-1 Δ*nalP*_GFP	BB-1 Δ*nalP* with insertion of *opaB*P$_H$-gfp into *hrtA* locus. Ery^R, Gm^R, Kan^R	This study
BB-1Δ*tpsA*	BB-1 derivative with *tpsA* and downstream *tpsCs* replaced by *kan.* Ery^R, Kan^R	[4]
BB-1Δ*mafAMGI-3*	BB-1 derivative with *mafA*$_{MGI\text{-}3}$ replaced by *kan.* Ery^R, Kan^R	[5]
HB-1	Derivative of H44/76; capsule locus replaced by *ery*, cc32. Ery^R	[6]
HB-1_GFP	HB-1 with insertion of *opaB*P$_H$-gfp into *hrtA* locus. Ery^R, Gm^R	This study

HB-1_RFP (a) (b)	HB-1 with insertion of *opaB*P_H-rfp into *hrtA* locus. Ery^R, Gm^R	This study
HB-1 Δ*nalP* (c)	HB-1; *nalP* replaced by *kan*. Ery^R, Kan^R	[7]
HB-1 Δ*nalP* ^{Rif} (d)	Rifampicin-resistant derivative of HB-1 Δ*nalP*. Ery^R, Kan^R, Rif ^{R}	This study
HB-1 Δ*nalP*_GFP	HB-1 Δ*nalP* with insertion of *opaB*P_H-gfp into *hrtA* locus. Ery^R, Kan^R, Rif^R	This study
HB-1 Δ*nalP*/NalP$^+$	HB-1; *nalP* replaced by *kan* and carrying pEN300. Ery^R, Kan^R, Cat^R	[7]
HB-1 Δ*nalP*/NalP$^+$_GFP	HB-1 Δ*nalP*/NalP$^+$ with insertion of *opaB*P_H-gfp into *hrtA* locus. Ery^R, Kan^R, Cam^R , Gm^R	This study
HB-1 Δ*autA*	HB-1; *autA* replaced by *kan*. Ery^R, Kan^R	[3]
HB-1 Δ*autA*/AutA$^+$	HB-1; *autA* replaced by *kan* and carrying pFPAutA. Ery^R, Kan^R, Cam^R	[3]
HB-1 Δ*autA*/AutA$^+$_GFP	HB-1 Δ*autA*/AutA$^+$ with insertion of *opaB*P_H-gfp into *hrtA* locus. Ery^R, Kan^R, Cam^R , Gm^R	This study
HB-3 Δ*nhbA* Δ*iga*	*nalP* and capsule locus deleted (both markerless knockout) with *nhbA* and *iga* replaced by *cat* and *kan*, respectively. Kan^R, Cam^R	[1]
HB-3 Δ*nhbA* Δ*iga*_GFP	HB-3 Δ*nhbA* Δ*iga* with insertion of opaBP_H-gfp into *hrtA locus*. Kan^R, Cam^R , Gm^R	This study
HB-1 Δ*pilE*	HB-1 derivative with *pilE* replaced by *Cam*. Ery^R, Cam^R	M. Bos
69cap	Derivative of 69; cc11; capsule locus replaced by *ery*. Ery^R	[1]
69cap_GFP	Derivative of 69cap with insertion of *opaB*P_H-gfp into *hrtA* locus. Ery^R, Gm^R	This study
2001044cap	Derivative of 2001044; cc11; capsule locus replaced by *cat*. Cam^R	[4]
2001044cap_RFP	Derivative of 2001044cap with insertion of *opaB*P_H-rfp into *hrtA* locus. Cam^R , Gm^R	This study
2070077cap	Derivative of 2070077; cc32; capsule locus replaced by *ery*. Ery^R	[1]
2070077cap_RFP	Derivative of 2070077cap with insertion of *opaB*P_H-rfp into *hrtA* locus. Ery^R, Gm^R	This study
2071066cap	Derivative of 2071066; cc32; capsule locus replaced by *ery*. Ery^R	[1]
2071066cap_GFP	Derivative of 2071066cap with insertion of *opaB*P_H-gfp into *hrtA* locus. Ery^R, Gm^R	This study
**Plasmids**		
pCRT_hrtA	Plasmid containing *hrtA* region. *kan*, *amp*	[8]
pEN300	Plasmid containing an nalP gene under an IPTG-inducible promoter. *ery, cat*	[9]
phrtA_gm_rfp	pCRT_hrtA containing *rfp* under *lac* promoter. *gm*, *kan*, *amp*	[3]
phrtA_gm_gfp	pCRT_hrtA containing *gfp* under *lac* promoter. *gm*, *kan*, *amp*	This study
pFPAutA	Plasmid containing an *autA* gene under an IPTG-inducible promoter. *ery, cat*	[3]
pIN_H	phrtA-gm-gfp containing *gfp* under *opaB*P_H promoter. *gm, kan, amp*	This study
pIN_M	phrtA-gm-gfp containing *gfp* under *opaB*P_M promoter. *gm*, *kan*, *amp*	This study
pIN_L	phrtA-gm-gfp containing *gfp* under *opaB*P_L promoter. *gm*, *kan*, *amp*	This study
pIN_{H-RED}	phrtA-gm-rfp containing *rfp* under *opaB*P_H promoter. *gm, kan, amp*	This study
mut 3.1	Plasmid containing a *gfp* gene under *lac* promoter	R. Nijland

[a] Strains underlined were used for CFU determination in mixed biofilms of HB-1 and BB-1 (a), HB-1 and BB-1Δ*nalP* (b), HB-1Δ*nalP* and BB-1 (c), HB-1Δ*nalP* and BB-1Δ*nalP (d)*, α14 and BB-1 *(e)* and α14 Δ*autA* and BB-1 *(f)*. *kan*, kanamycin-resistance cassette; *amp*, ampicillin-resistance cassette; *gm*, gentamicin-resistance cassette ; *cat*, chloramphenicol-resistance cassette; cc, clonal complex. UU, Utrecht University collection.

References

1. Arenas J., Nijland R., Rodriguez F.J., Bosma T.N., Tommassen J. (2013). Involvement of three meningococcal surface-exposed proteins, the heparin-binding protein NHBA, the α-peptide of IgA protease and the autotransporter protease NalP, in initiation of biofilm formation. *Mol. Microbiol*. 87, 254-268.

2. Schoen C., Blom J., Claus H., Schramm-Glück A., Brandt P., Müller T., et al. (2008). Whole-genome comparison of disease and carriage strains provides insights into virulence evolution in *Neisseria meningitidis. Proc. Natl. Acad. Sci. U.S.A.*105, 3473-3478.

3. Arenas J., Cano S., Nijland R., van Dongen V., Rutten L., van der Ende A., et al. (2015). The meningococcal autotransporter AutA is implicated in autoaggregation and biofilm formation. *Environ. Microbiol*. 17, 1321-1337.

4. Arenas J., Schipper K., van Ulsen P., van der Ende A., Tommassen J. (2013). Domain exchange at the 3' end of the gene encoding the fratricide meningococcal two-partner secretion protein A. *BMC Genomics* 14, 622.

5. Arenas, J., de Maat, V., Catón, L., Krekorian, M., Herrero, J.C., Ferrara, F., Tommassen J. (2015). Fratricide activity of MafB protein of N. meningitidis strain B16B6. *BMC Microbiol*.15, 156.

6. Bos M.P., Tommassen J. (2005). Viability of a capsule- and lipopolysaccharide-deficient mutant of *Neisseria meningitidis*. *Infect. Immun*.73, 6194-6197.

7. van Ulsen P., van Alphen L., ten Hove J., Fransen F., van der Ley P., Tommassen J. (2003). A Neisserial autotransporter NalP modulating the processing of other autotransporters. *Mol. Microbiol*. 50, 1017-1030.

8. Roussel-Jazédé V., Grijpstra J., van Dam V., Tommassen J., van Ulsen P. (2013). Lipidation of the autotransporter NalP of *Neisseria meningitidis* is required for its function in the release of cell-surface-exposed proteins. *Microbiology* 159, 286-295.

9. van Ulsen P., van Alphen L., ten Hove J., Fransen F., van der Ley P., Tommassen J. (2003). A Neisserial autotransporter NalP modulating the processing of other autotransporters. *Mol Microbiol*. 50, 1017-1030.
