# Supplementary material for: Interstrain Cooperation in Meningococcal Biofilms: Role of Autotransporters NalP and AutA
Source: Front Microbiol. 2017 Mar 22;8:434. doi: 10.3389/fmicb.2017.00434 (PMC5360712; doi:10.3389/fmicb.2017.00434)
Supplement: Supplementary file 2 [file Table2.PDF]

TABLE S2 Primers used in this study

Primer name	Sequence (5´→ 3´)	Goal
Fwmut3.1MluI	CGCGCGACGCGTGCGCAACGCAATTAATGTGAGTT	amplify *lac-gfp*
Remut3.1PpuMI	GCGCGCAGGACCCCTCAGTACAATCTGCTCTGA	
FwGenPpuMI	GCGCGCAGGACCCAGACGCACACCGTGGAAA	amplify *gen*
RvGenPpuMI	GCGCGCAGGACCCAGCGGCGTTGTGACAATTT	
Fw GFP-NheI	GCGCGCGCTAGCATGCGTAAAGGAGAAGAACT	amplify *gfp*
Rv GFP-Van91I	GCGCGCCCAGGTAGTGGCCCCTCAGTACAATCTGCTC	
Fwopa-MluI	GCGCGCACGCGTGGAATGACGGCGGAAAGATG	amplify *opaB*P_M
Rvopa-NheI	GCGCGCGCTAGCCTCCTTATTCGGTTTAACCG	
Fwopa-MluI	GCGCGCACGCGTGGAATGACGGCGGAAAGATG	amplify *opaB*P_L
RvopaL-SmaI	GCGCGCCCCGGGAAAAGATTTTCACTGATGTT	
Fwopa-MluI	GCGCGCACGCGTGGAATGACGGCGGAAAGATG	amplify *opaB*P_H
RvopaH-SmaI	GCGCGCCCCGGGAAGGGCGGATTATATCGGGT	

Sequences for restriction sites, which are also included in primer names, are underlined.
