# Supplementary material for: Interstrain Cooperation in Meningococcal Biofilms: Role of Autotransporters NalP and AutA
Source: Front Microbiol. 2017 Mar 22;8:434. doi: 10.3389/fmicb.2017.00434 (PMC5360712; doi:10.3389/fmicb.2017.00434)
Supplement: Supplementary file 3 [file Image1.PDF]

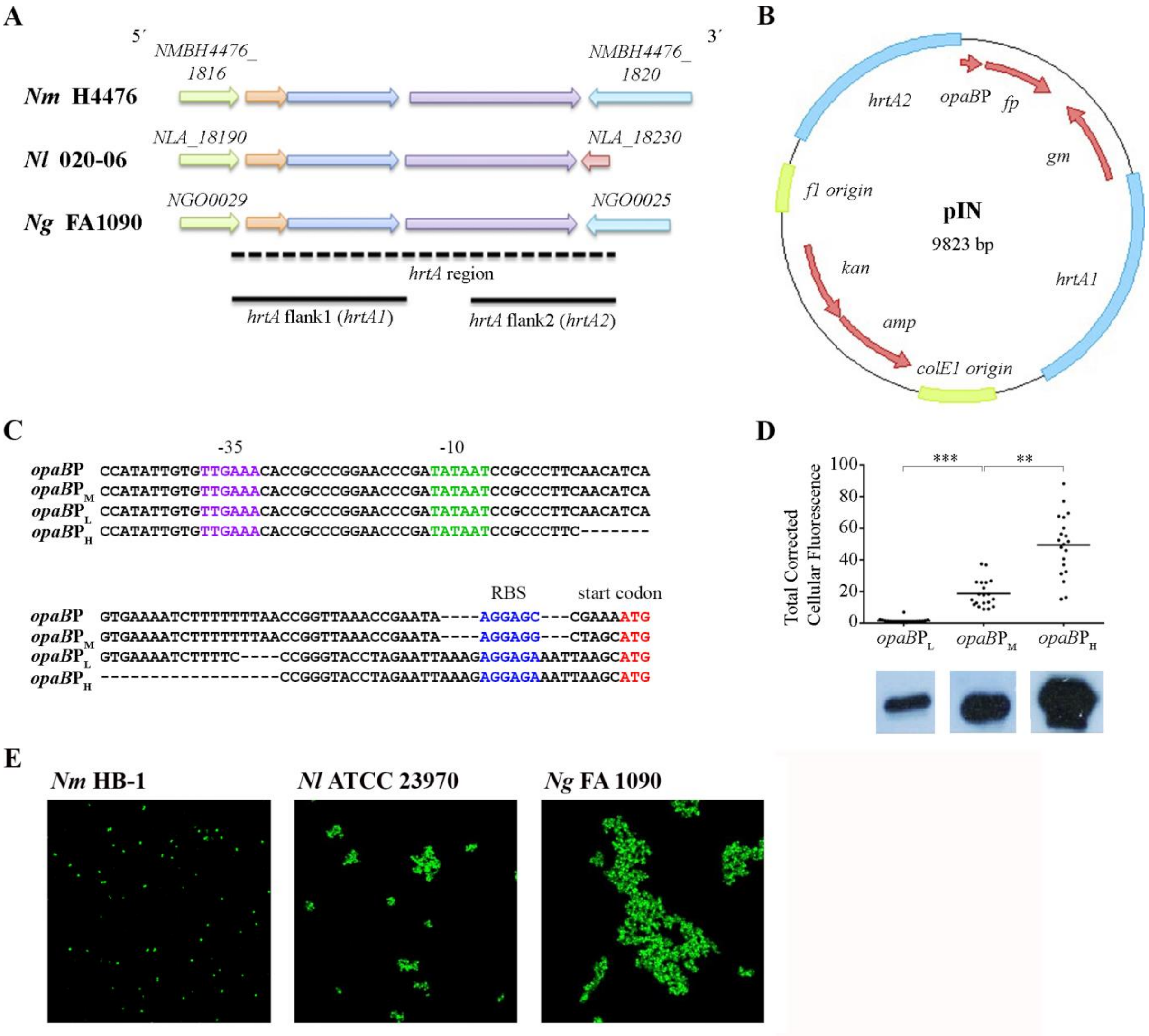

Figure S1 | Characteristics of pIN plasmids for engineering fluorescent neisseriae. (**A**) Genomic context of *hrtA* loci in *Neisseria* spp. The DNA segments labelled *hrtA*$_1$ and *hrtA*$_2$ were used in pIN plasmids to provide targets for homologous recombination. Genes are represented by arrows and those with the same colour contain > 90% sequence identity between species. From left to right, the genes encode: green, orotate phosphoribosyl transferase (PyrE); orange, a conserved hypothetical protein; blue, N-acetylglutamate synthase; purple, a putative peptidase; cyan, a putative AraC-like transcriptional regulator; pink, a putative rhodanese. (**B**) Map of pIN plasmids containing the *hrtA*$_1$ and *hrtA*$_2$

segments, an *opaB* promoter variant (*opaB*P) that controls the expression of a gene encoding a fluorescent protein (*fp*), and three antibiotic-resistance cassettes, i.e. *gm*, *kan* and *amp*, providing resistance to gentamicin, kanamycin and ampicillin, respectively. Genes and the *opaB*P are indicated by red arrows, the *hrtA* regions by blue boxes, and the origins of replication by green boxes. (**C**) Alignment of the *opaB*P from *N. gonorrhoeae* strain FA 1090 and the H, M and L derivatives used in this study. The -35 and -10 hexamers, Shine-Dalgarno sequence (RBS) and the start codon for the fluorescent proteins are indicated with purple, green, blue and red letters, respectively. (**D**) Quantification of the relative fluorescence of derivatives of *N. meningitidis* HB-1 expressing GFP under the control of H, M and L *opaB*P variants. The graft shows the total corrected cellular fluorescence of 20 individual cells calculated using ImageJ software; the resulting mean is indicated with a line. Statistically significant differences are marked with two ($P < 0.005$) or three asterisks ($P < 0.0005$) (unpaired t-test). The lower part shows a Western blot of whole cell lysates of HB-1 derivatives expressing GFP probed with anti-GFP antibodies. The signals for each sample were obtained on the same membrane but separated for clarity. (**E**) Microscopy images of *Nm*, *Nl* and *Ng* strains transformed with plasmid pIN_H grown in liquid cultures.
