# Supplementary material for: Interstrain Cooperation in Meningococcal Biofilms: Role of Autotransporters NalP and AutA
Source: Front Microbiol. 2017 Mar 22;8:434. doi: 10.3389/fmicb.2017.00434 (PMC5360712; doi:10.3389/fmicb.2017.00434)
Supplement: Supplementary file 4 [file Image2.PDF]

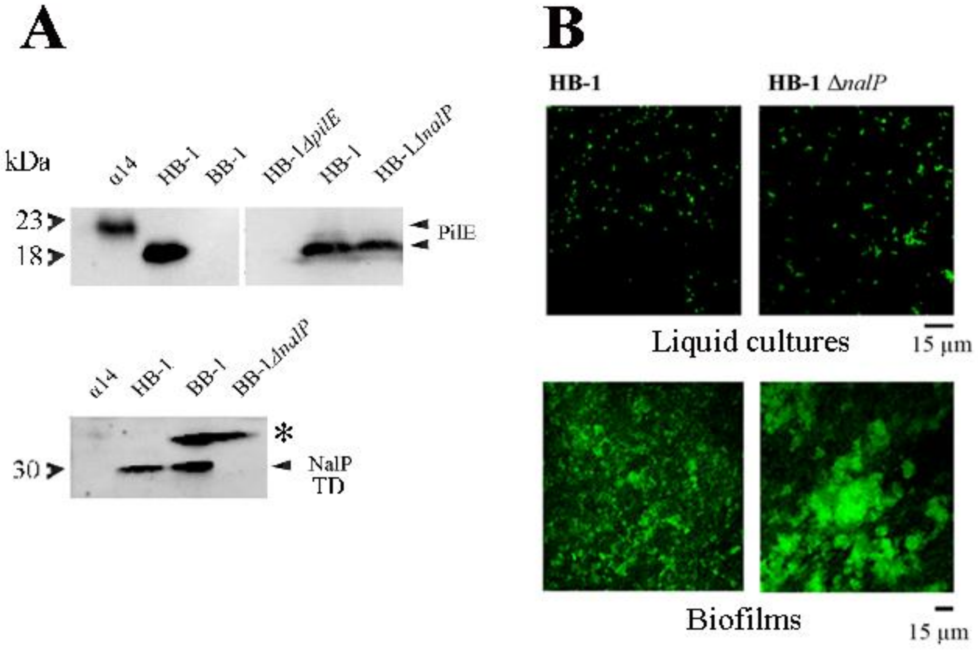

Figure S2 | Influence of NalP on microcolony formation in biofilms. (A) Western blotting of whole cell lysates of α14, BB-1, and of HB-1 and its *pilE* and *nalP* mutant derivatives probed with a monoclonal antibody directed against PilE (top panel). The lower panel shows a Western blot of whole cell lysates of α14, HB-1, and BB-1 and its *nalP* mutant derivative probed with an antiserum directed against the translocator domain (TD) of NalP. The position of a cross-reacting band in the lysates of BB-1 and its *nalP* mutant derivative is indicated with an asterisk. In both blots, equal quantities of lysates, based on the OD_{550} of the cultures, were applied. Apparent molecular weights of relevant bands are indicated at the left side. **(B)** Microscopy images of HB-1 and its *nalP* mutant derivative grown in liquid cultures and of 15-h old biofilms.
