# Supplementary material for: Interstrain Cooperation in Meningococcal Biofilms: Role of Autotransporters NalP and AutA
Source: Front Microbiol. 2017 Mar 22;8:434. doi: 10.3389/fmicb.2017.00434 (PMC5360712; doi:10.3389/fmicb.2017.00434)
Supplement: Supplementary file 5 [file Image3.PDF]

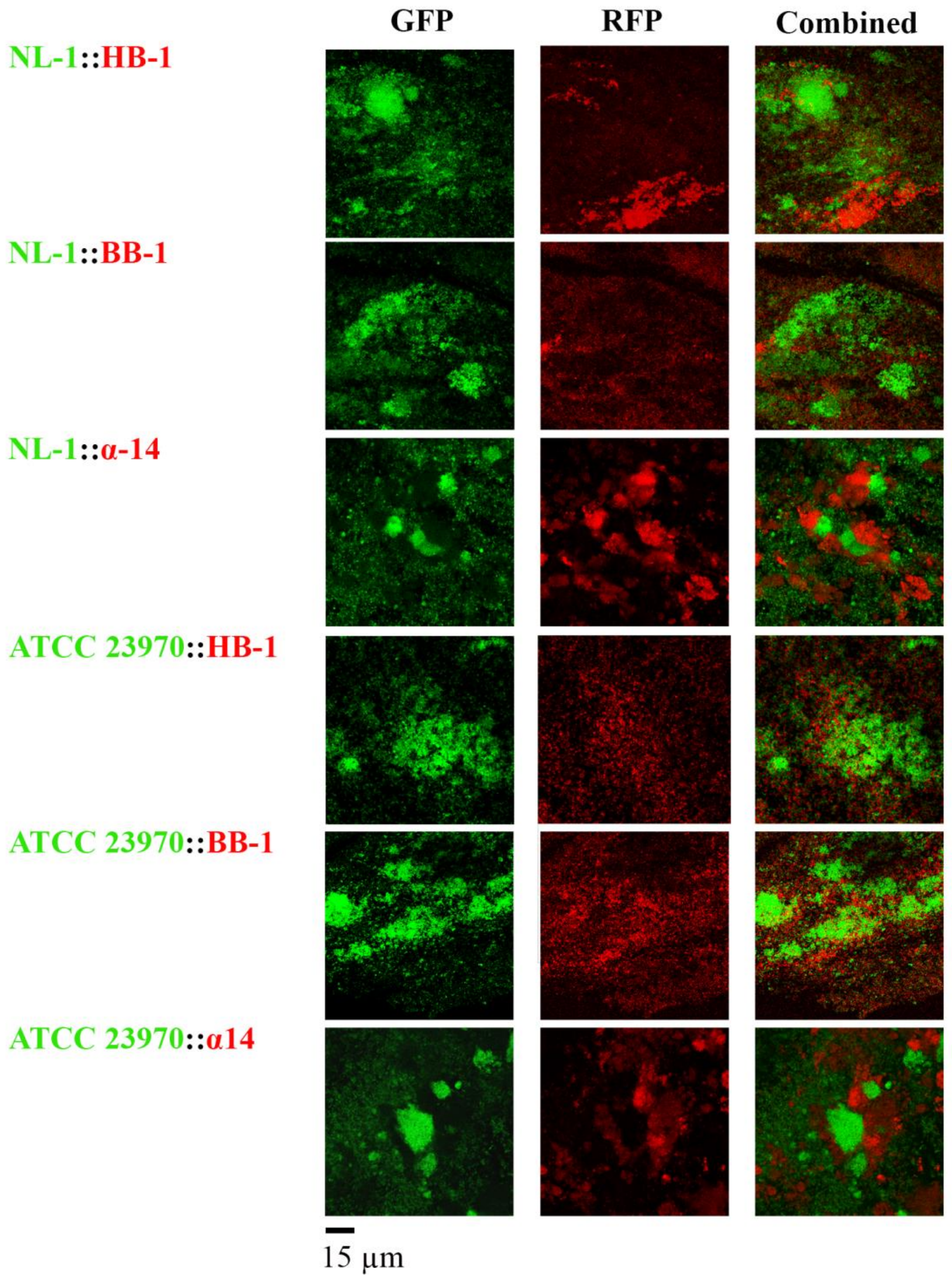

Figure S3 | Interbacterial interactions between *Nm* and *Nl* in biofilms. Combinations of *Nm* strains HB-1, BB-1, and α14 with *Nl* strains NL-1 and ATCC23970. Strain names at the left are shown in green or red, reflecting the expression of GFP or RFP, respectively. Individual and combined fluorescence are displayed.
