# Supplementary material for: Interstrain Cooperation in Meningococcal Biofilms: Role of Autotransporters NalP and AutA
Source: Front Microbiol. 2017 Mar 22;8:434. doi: 10.3389/fmicb.2017.00434 (PMC5360712; doi:10.3389/fmicb.2017.00434)
Supplement: Supplementary file 6 [file Image4.PDF]

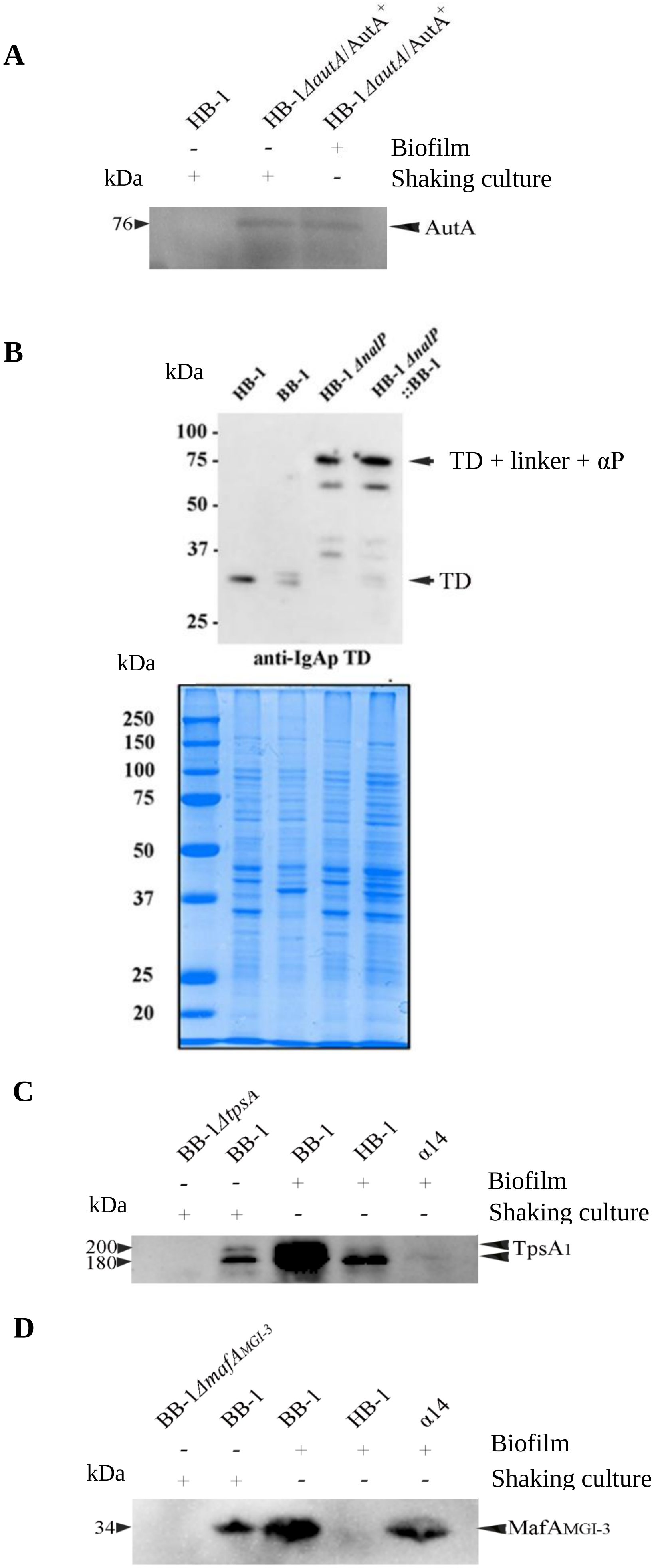

A
HB-1
HB-1ΔautA/AutA×
HB-1ΔautA/AutA×
Biofilm
Shaking culture
kDa
76
AutA
B
kDa
HB-1
BB-1
HB-1 ΔnalP
HB-1 ΔnalP ::BB-1
100
75
50
37
25
TD + linker + αP
TD
anti-IgAp TD
kDa
250
150
100
75
50
37
25
20
C
BB-1ΔtpsA
BB-1
BB-1
HB-1
α14
Biofilm
Shaking culture
kDa
200
180
TpsA1
D
BB-1ΔmafAMGI-3
BB-1
BB-1
HB-1
α14
Biofilm
Shaking culture
kDa
34
MafAMGI-3

Figure S4 | Production of proteins in biofilms. (A) Synthesis of AutA in biofilms of HB-1 carrying plasmid pFPAutA. Western blot of whole cell lysates of HB-1 and HB-1Δ*autA* carrying pFPAutA (HB-1Δ*autA*/AutA^{+}) extracted from 6-h old shaking cultures and from 15-h old biofilms both grown in the presence of IPTG. **(B)** Secreted NalP from BB-1 does not cleave the αP from the cell surface of HB-1Δ*nalP* in dual-strain biofilms. The upper panel shows a Western blot of whole cell lysates extracted from 15-h old biofilms. The blot was probed with an antiserum directed against the translocator domain (TD) of IgA protease. In the absence of NalP, the αP remains fused to the TD by a linker domain. Different fragments of IgA protease are labelled at the right side of the blot. Note that also in the presence of BB-1, which produces NalP, the αP remains associated to the TD in the HB-1Δ*nalP* cells. In the lower panel, proteins in the samples were visualized by Coomassie blue staining of a gel. **(C)** TpsA1 is expressed and secreted in biofilms. A Western blot containing concentrated proteins from supernatants of 6-h old shaking cultures and from 15-h old biofilms was probed with an antiserum raised against the conserved TPS domain of TpsA1. TpsA1 proteins are processed after secretion, resulting in the detection of two different forms (van Ulsen *et al*., 2008). **(D)** $MafA_{MGI\text{-}3}$ is synthetized in biofilms. A Western blot containing whole cell lysates from 6-h old shaking cultures and 15-h old biofilms was probed with an antiserum raised against $MafA_{MGI\text{-}3}$ of strain BB-1. All three strains contain one $\textit{mafA}_{MGI\text{-}3}$ gene but the gene is disrupted in *N. meningitidis* H44/76. In panels A, C and D, only the relevant parts of the blots are shown and the apparent molecular weights of the bands are indicated at the left. In panel B, the positions of molecular-mass marker proteins are shown at the left. Equal quantities of cell lysates (panels A, B and D) or extracellular media (panel C) were applied in each lane based on the OD_{550} of the cultures or resuspended biofilms.
