# Supplementary material for: Interstrain Cooperation in Meningococcal Biofilms: Role of Autotransporters NalP and AutA
Source: Front Microbiol. 2017 Mar 22;8:434. doi: 10.3389/fmicb.2017.00434 (PMC5360712; doi:10.3389/fmicb.2017.00434)
Supplement: Supplementary file 7 [file Image5.PDF]

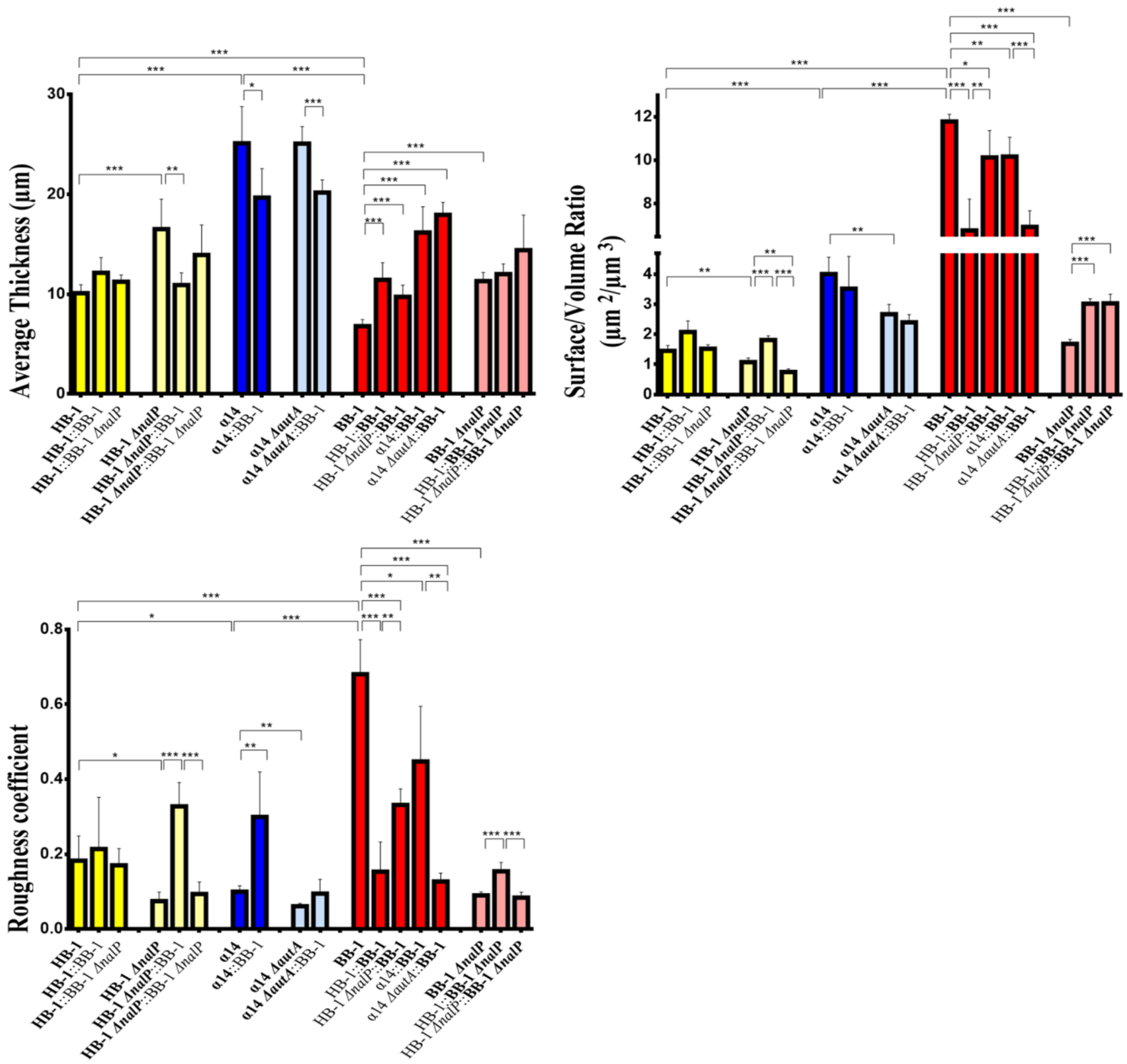

Figure S5 | Characteristics of single- and dual-strain biofilms of *Nm* strains. The average thickness, surface/volume ratio, and roughness coefficient of biofilms formed in TSB medium were calculated for each strain using COMSTAT. The strains present in the consortium are shown at the bottom of each panel. The data shown are for the strain indicated in bold. Statistically significant differences are marked with one ($P < 0.05$), two ($P < 0.005$) or three asterisks ($P< 0.0005$) (unpaired t-test). Results are means and standard deviations of three independent experiments.
